# Site-Specific Glycosylation of Virion-Derived HIV-1 Env Is Mimicked by a Soluble Trimeric Immunogen

**DOI:** 10.1016/j.celrep.2018.07.080

**Published:** 2018-08-24

**Authors:** Weston B. Struwe, Elena Chertova, Joel D. Allen, Gemma E. Seabright, Yasunori Watanabe, David J. Harvey, Max Medina-Ramirez, James D. Roser, Rodman Smith, David Westcott, Brandon F. Keele, Julian W. Bess, Rogier W. Sanders, Jeffrey D. Lifson, John P. Moore, Max Crispin

**Affiliations:** 1 Oxford Glycobiology Institute, Department of Biochemistry, University of Oxford, Oxford OX1 3QU, UK; 2 Chemistry Research Laboratory, Department of Chemistry, University of Oxford, 12 Mansfield Road, OX1 3TA, UK; 3 Biological Sciences and the Institute for Life Sciences, University of Southampton, Southampton SO17 1BJ, UK; 4 Department of Microbiology and Immunology, Weill Cornell Medical College, Cornell University, New York, NY, USA; 5 AIDS and Cancer Virus Program, Leidos Biomedical Research, Frederick National Laboratory for Cancer Research, Frederick, MD, USA; 6 Department of Medical Microbiology, Academic Medical Center, University of Amsterdam, Amsterdam, the Netherlands; 7 Target Discovery Institute, Nuffield Department of Medicine, University of Oxford, Roosevelt Drive, Oxford OX3 7FZ, UK

**Keywords:** HIV, glycosylation, mass spectrometry, envelope glycoprotein

## Abstract

Many broadly neutralizing antibodies (bnAbs) against HIV-1 recognize and/or penetrate the glycan shield on native, virion-associated envelope glycoprotein (Env) spikes. The same bnAbs also bind to recombinant, soluble trimeric immunogens based on the SOSIP design. While SOSIP trimers are close structural and antigenic mimics of virion Env, the extent to which their glycan structures resemble ones on infectious viruses is undefined. Here, we compare the overall glycosylation of gp120 and gp41 subunits from BG505 (clade A) virions produced in a lymphoid cell line with those from recombinant BG505 SOSIP trimers, including CHO-derived clinical grade material. We also performed detailed site-specific analyses of gp120. Glycans relevant to key bnAb epitopes are generally similar on the recombinant SOSIP and virion-derived Env proteins, although the latter do contain hotspots of elevated glycan processing. Knowledge of native versus recombinant Env glycosylation will guide vaccine design and manufacturing programs.

## Highlights

• HIV envelope glycans are central features of broadly neutralizing antibody epitopes
• Recombinant mimetics of the HIV virus trimer are vaccine candidates
• Glycosylation of a leading trimer immunogen is similar to that of infectious virus

## Graphical Abstract

**Figure:**
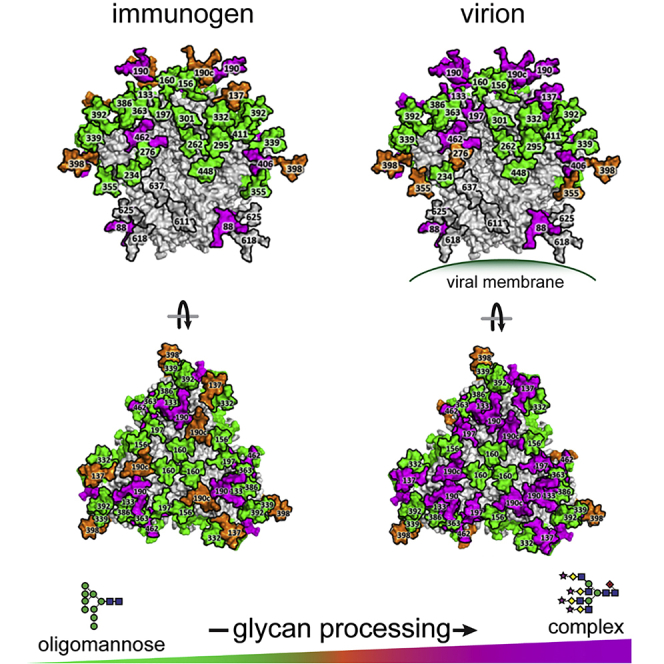

## Teaser

Struwe et al. present site-specific analyses of N-glycosylation sites on HIV-1 envelope glycoproteins from an infectious virus and a recombinant trimer mimic. The structural and antigenic details of the glycan shield will be valuable for designing next-generation immunogens and understanding virus neutralization by broadly active antibodies.

## Introduction

Fundamental features of retroviral biology hinder the development of an effective vaccine against HIV-1. A rapid mutation rate creates profound antigenic diversity that can only be countered by immune responses of exceptional breadth. A barrier to a neutralizing antibody (nAb)-based vaccine is the dense coat of glycans covering the surface of the envelope glycoprotein (Env) spike that restricts the immunogenicity of Env proteins and limits the binding of antibodies that are elicited.

The HIV-1 Env glycans were long considered to be merely a passive shield; the number of individual glycan sites and their precise locations on Env evolve during infection as a dynamic response to nAb selection pressures (Moore et al., 2012, Sagar et al., 2006, Wei et al., 2003). However, various broadly neutralizing antibodies (bnAbs) of substantial potency that do not merely penetrate the glycan shield but also recognize elements of it can be isolated from infected individuals. Passively administered bnAbs protect macaques or humanized mice against virus challenge with an efficiency that is generally predicted by *in vitro* neutralization titers (Pegu et al., 2017, van Gils and Sanders, 2014). The existence of bnAbs combined with an understanding of their properties together underpin vaccine designs that are intended to induce similar antibodies. In general, these concepts involve producing recombinant mimics of the native, virion-associated Env spike that present multiple bnAb epitopes and/or their predicted human germline precursors (Sanders and Moore, 2017, Stamatatos et al., 2017).

The most commonly used HIV-1 Env immunogen design platform is based on SOSIP.664 trimers (SOSIP), recombinant proteins rendered soluble by truncation at position 664 and engineered for improved stability by the addition of a disulfide bond (SOS) and an isoleucine-to-proline mutation (IP) (Binley et al., 2000, Sanders et al., 2013, Sanders and Moore, 2017). The prototype and most widely studied SOSIP trimer is derived from the BG505 clade A pediatric isolate (Goo et al., 2014). BG505 SOSIP.664 trimers have Env spike-mimicking antigenicity and structural properties (Dey et al., 2018, Sanders et al., 2013). They have now been produced in gram quantities in Chinese hamster ovary (CHO) cells under Current Good Manufacturing Practice (CGMP) conditions for human trials (Dey et al., 2018). Because glycans both shield and form bnAb epitopes (Crispin et al., 2018) and because viral neutralization by bnAbs is influenced by glycan heterogeneity (McCoy et al., 2015), it is of substantial relevance to characterize them on both the viral target and HIV-1 Env immunogens. The unusually high surface density of glycans on these proteins restricts the extent to which multiple individual sites are processed within the endoplasmic reticulum and the Golgi apparatus (Pritchard et al., 2015b). Env processing is restricted on two levels. Thus, monomeric gp120 subunits and gp140 pseudotrimers that are predominantly in non-native configurations carry a range of highly processed, complex-type glycans together with a smaller population of unprocessed oligomannose-type glycans (Man_5–9_GlcNAc_2_, hereafter referred to as Man5–9) (Bonomelli et al., 2011, Go et al., 2013, Pritchard et al., 2015c). Additional influences were revealed when SOSIP trimers were found to bear substantially increased levels of oligomannose-type glycans (Behrens and Crispin, 2017, Behrens et al., 2016, Cao et al., 2017, Pritchard et al., 2015c). The packing of gp120 subunits into native-like trimers further shapes Env glycosylation because key glycan-processing enzymes have a limited ability to encounter their substrates; access is sterically hindered by nearby glycan and protein elements (Behrens and Crispin, 2017, Crispin et al., 2018). As similarly high levels of oligomannose-type glycans are present on native virion Env, the antigenic and structural homologies between SOSIP trimers and Env spikes are further reflected by their glycan profiles (Bonomelli et al., 2011, Panico et al., 2016, Ringe et al., 2015). However, the complex-type glycans found at multiple sites on Env proteins are not constrained in the same way as oligomannose sites but are instead influenced by the prevailing secretory environment in a cell-specific manner (Ringe et al., 2015). This factor is relevant to immunogen design because certain complex-type glycans can also contribute to bnAb epitopes and, perhaps, to immunogenicity (Andrabi et al., 2017, Blattner et al., 2014, Crispin et al., 2018, Gristick et al., 2016, Mouquet et al., 2012).

Overall, site-specific analyses have revealed a mosaic nature to the distribution of mannose moieties across the surface of SOSIP trimers (Behrens et al., 2016, Cao et al., 2017, Crispin et al., 2018). Regions of oligomannose-type glycans located at equivalent positions on the gp120 monomer and SOSIP trimer constitute the intrinsic mannose patch (IMP) (Behrens and Crispin, 2017, Cao et al., 2017), whereas other such glycans that are trimer specific are designated the trimer-associated mannose patch (TAMP) (Behrens and Crispin, 2017, Doores et al., 2010). Analysis of cell line-derived HIV-1 BaL virions (Panico et al., 2016) and membrane-tethered forms of Env have suggested that there could be factors leading to divergent glycosylation compared to soluble trimers (Go et al., 2017).

Here, we directly compared the glycosylation of Env derived from the BG505.T332N-LAI virus, produced in a human lymphoid cell line, with the sequenced-matched BG505 SOSIP.664 soluble trimers expressed in CHO or 293F cells. We quantified the abundance of oligomannose and complex N-linked glycans on the gp120 and gp41 subunits by chromatographic methods and characterized glycan structures by ion-mobility-tandem mass spectrometry (IM-MS/MS). To characterize site-specific differences, we used complimentary approaches involving the comparison of intact and glycosidase-treated glycopeptides. Sufficient gp120, but not gp41, could be isolated from virions for glycopeptide analysis. We found similarities in how the glycans on virion and SOSIP.664 glycoproteins were processed, but also some divergences; glycan sites that were only partially processed on the SOSIP.664 trimers were more processed to complex-type structures on the virus-derived Env proteins. Overall, this information on the glycan-dependent or glycan-influenced bnAb epitopes present on infectious HIV-1 virions will facilitate the continued development of Env immunization strategies that are intended to eventually elicit bnAb-like antibodies.

## Results and Discussion

### Preparation of HIV-1 Virion-Derived Env

An infectious stock of HIV-1 BG505.T332N-LAI was derived by transfecting 293T cells with the infectious molecular clone (IMC) (Figure S1). The IMC Env glycoproteins have gp120- and gp41-ectodomain subunits derived from the primary R5 tier-2 clade A BG505 week 6 isolate, but with the N332 glycan knocked in to mimic the BG505 SOSIP.664 soluble trimer sequence (Medina-Ramírez et al., 2017, Sanders et al., 2013). The transfection stock was used to infect the CD4^+^, CCR5^+^ A66-R5 T cell line to provide the large amount of virus required for glycan analysis (Figure S1; Table S1).

A 1.9-mL aliquot of the 1,000-fold concentrated virus stock was processed by high-performance liquid chromatography (HPLC) (Figure S1C). The fractions containing gp120 and gp41 subunits were then run on an SDS-PAGE gel (Figures S1D and S1E). The gp120- and gp41-containing bands were excised from the gel for the analysis of released glycans by ultra-high-performance liquid chromatography (UPLC) and MS. A separate 2.7 mL aliquot of the virus stock was then purified (Figures S1F and S1G). The pooled gp120 fractions (∼70 μg) were subsequently used for site-specific glycan analyses.

### Relative Abundance of Oligomannose- versus Complex-type Glycans

The purified gp120 and gp41 gel bands shown in Figures S1D and S1E were subjected to in-gel peptide-N-glycosidase F (PNGase F) digestion, which removes both oligomannose- and complex-type glycans. The relative amounts of oligomannose- versus complex-type glycans on the virion gp120 and gp41 subunits were compared to recombinant BG505 SOSIP.664 trimers that were produced either transiently in 293F cells and purified via the PGT151 bnAb or under CGMP conditions in a stable CHO cell line and purified via 2G12/SEC (Dey et al., 2018, Ringe et al., 2015). These comparisons are relevant because initial characterizations of the BG505 trimer glycans used 293F cell-produced proteins, while human clinical studies will be performed with CGMP-grade trimers derived from CHO cells (Dey et al., 2018). Furthermore, the goal of the clinical studies is to elicit nAbs that recognize functional Env proteins present on infectious virions that are produced predominantly in human lymphoid cells.

**Figure 1:**
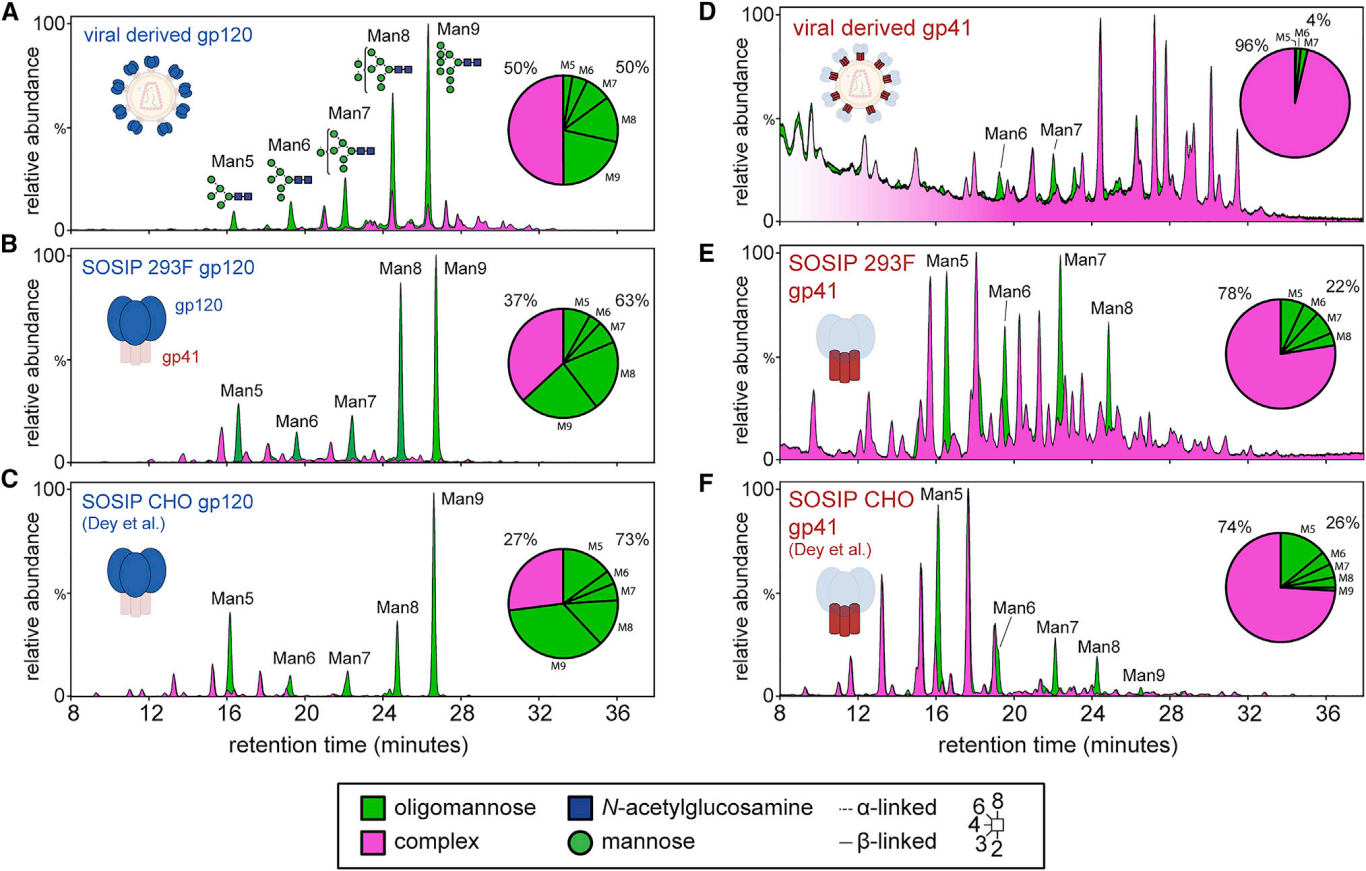
UPLC Analysis of N-Glycans from gp120 and gp41 (A) UPLC chromatogram of N-glycans from BG505.T332N-LAI gp120 gel bands. Peaks sensitive to endoglycosidase H (endoH) digestion (green) represent oligomannose-type glycans. Peaks resistant to endoH (magenta) represent complex-type glycans. Pie charts depict the quantification of oligomannose and complex-type glycans in each sample. Peaks corresponding to oligomannose-type glycans are annotated with the number of mannose residues (Man5–9). (B) UPLC chromatogram of N-glycans released from gp120 from BG505 SOSIP.664 trimers produced in 293F cells. (C) Reproduction of the UPLC analysis of N-glycans released from BG505 SOSIP.664 trimers produced in CHO cells (Dey et al., 2018). (D–F) (D), (E), and (F) show data for gp41 and correspond to gp120 (A), (B), and (C), respectively. See also Figure S1 and Table S2.

Differences between the Env forms were quantified using a UPLC-based analytical procedure whereby differential sensitivity to endoglycosidase H (endoH) reveals the abundances of oligomannose- and hybrid-type glycans (Figure 1 [green chromatograms]) and complex-type glycans (Figure 1 [magenta chromatograms]). The N-glycans on the virion-derived gp120 proteins were 50% oligomannose type and 50% complex type (Figure 1A), whereas the gp120 subunits of the SOSIP.664 trimers were enriched for oligomannose-type structures: 63% for the 293F cell product (Figure 1B) and 73% for CHO (Figure 1C) (Dey et al., 2018). There were also differences in the oligomannose-type structures present on the three sources of gp120 (Figure 1; Table S2). For example, Man9 and Man8 moieties were prominent in the SOSIP.664 gp120 spectra. Similarly, virion gp41 had a lower overall content of oligomannose-type N-glycans (4%; Figure 1D) compared to gp41 from SOSIP.664 trimers (22% [293F] and 26% [CHO]; Figures 1E and 1F, respectively). We conclude that glycans on the viral Env proteins are somewhat more processed than those on the recombinant trimers.

Larger and more elaborate, complex-type glycans were also relatively abundant on the virus-derived gp120 and gp41 subunits, compared to SOSIP.664 (Figure 1 [magenta chromatograms]). This is exemplified by N-glycans containing either α2-3- or α2-6-linked terminal sialic acids. Overall, the extent of gp120 sialylation was higher for BG505.T332N-LAI virions (16%) than for 293F-derived BG505 SOSIP.664 trimers (5%), while for the gp41 subunits the corresponding values were 26% and 8%, respectively. The ratio of α2-6/α2-3 sialic acids on the gp41 subunits was 1.8 for virions and 0.8 for 293F-derived trimers (Table S2), a finding consistent with earlier observations that α2-6-linked sialic acid moieties were prevalent on peripheral blood mononuclear cell (PBMC)-derived virion gp41 (Pritchard et al., 2015a). However, compared to gp41, the gp120 proteins from all three Env proteins contained a smaller population of sialic acid-containing glycans (Table S2). This observation may reflect a generally lower efficiency of glycan processing on gp120 subunits than on gp41 subunits, irrespective of the Env protein source.

### Analysis of Glycans by Ion-Mobility-Mass Spectrometry

**Figure 2:**
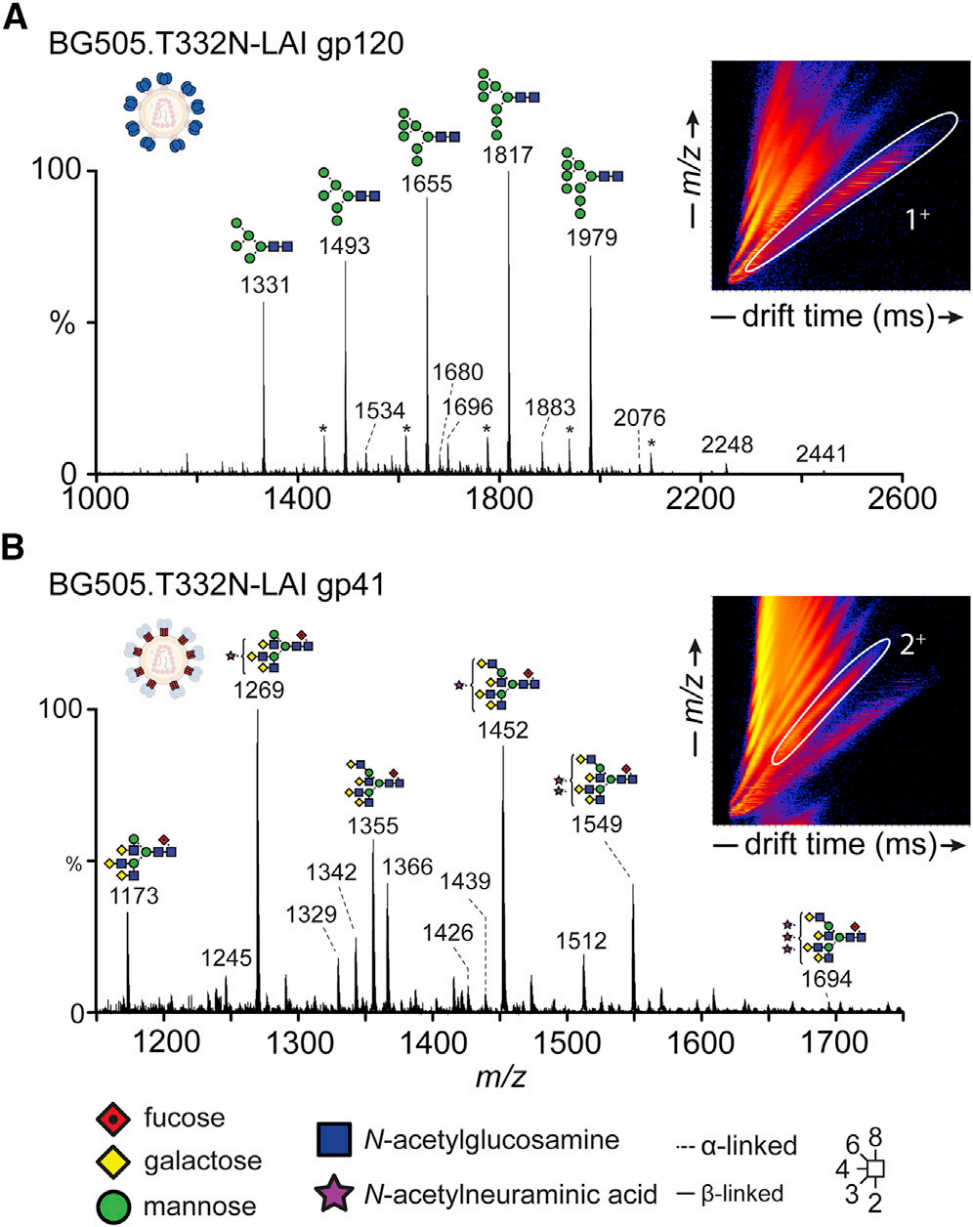
Ion-Mobility-Mass Spectrometry Analysis of BG505.T332N-LAI Virion-Derived gp120 and gp41 N-Glycans (A) Mobility-extracted singly charged negative ions from BG505.T332N-LAI gp120. The corresponding singly charged ions ([M+H_2_PO_4_]^−^) are encircled in white in the ion mobility drift plot (inset). The series of carbohydrate ions marked with an asterisk is formed by the additional adduction of sodium phosphate to the main oligomannose ions. (B) Mobility-extracted doubly charged negative ions from BG505.T332N-LAI gp41. The corresponding doubly charged ions (2^+^) are encircled in white in the ion mobility drift plot. See also Figures S2–S4.

To guide subsequent glycopeptide analyses, we performed IM-MS on a separate, unlabeled aliquot of glycans released from gp120 and gp41 subunits. Comparing the BG505.T332N-LAI virion Env and the 293F-derived SOSIP.664 trimers allows fine structure determinations, namely glycan linkage and branching and the compositions of individual glycans. In this way, we created extensive, sample-specific glycan libraries that were then used to search the corresponding site-specific data (Figure 2). In addition, we used collision-induced dissociation (CID) fragmentation in negative mode to obtain diagnostic fragment ions of the fine structures of the detected glycans (Figures S2, S3, and S4). We found that the oligomannose-type structures on gp120 from the BG505.T332N-LAI virus (Figure 2A) and the 293F cell-derived BG505 SOSIP.664 trimers (Figures S2 and S3) were highly similar. The major non-oligomannose glycans on BG505.T332N-LAI virion gp120 were various hybrid-type structures—glycans on which some arms have a terminal mannose—while others are more extensively processed (Figures 2 and S4A). Consistent with the overall trend detected by UPLC (Figure 1), the ion intensities of singly and doubly charged ions corresponding to more elaborate complex-type glycans were significantly higher in the virion-derived material compared to those of SOSIP.664 trimers (Figures 2, S3, and S4).

### Complex-type Structures on gp41

The glycans present on BG505.T332N-LAI virion gp41 were primarily core fucosylated, tri- and tetra-antennary species containing a variable number of terminal sialic acid residues (Figure 2B). Consistent with the UPLC analysis, larger complex-type glycans were more abundant on gp41 from virions than from 293F cell-derived trimers (Figure S3B), although generally similar structures were present in both cases. One caveat is that we have not controlled for the producer cell, which is not the same for the BG505.T332N-LAI virion (A66-R5 lymphoid cells) and the BG505 SOSIP.664 (CHO or 293F cells) Env proteins. Even so, we can reasonably conclude that membrane localization does not lead to a sterically driven increase in oligomannose-type glycans. Membrane tethering may, however, influence the degree of branching and elaboration of complex-type glycans by driving proximity to processing enzymes and influencing the kinetics of egress through the secretory system.

### Quantifying the Amounts of Underprocessed Glycans across gp120

Understanding how HIV-1 Env proteins are glycosylated and defining what glycoforms are present are important because almost the entire surface of the trimer contributes to bnAb epitopes (Burton and Hangartner, 2016, Crispin et al., 2018). The methodology paradigm for defining which type of glycan (i.e., complex versus oligomannose) is present at each potential glycosylation site involves a proteomic-based analysis of glycopeptides that have been enzymatically treated to create mass modifications that are specific to each glycan type (Cao et al., 2017). This method is highly robust for determining the ratio of oligomannose-type versus complex glycans, even when only low amounts of sample are available. We therefore sought to apply this method to the gp120 subunits of the BG505.T332N-LAI virus, using the HPLC-purified material shown in Figures S1F and S1G.

**Figure 3:**
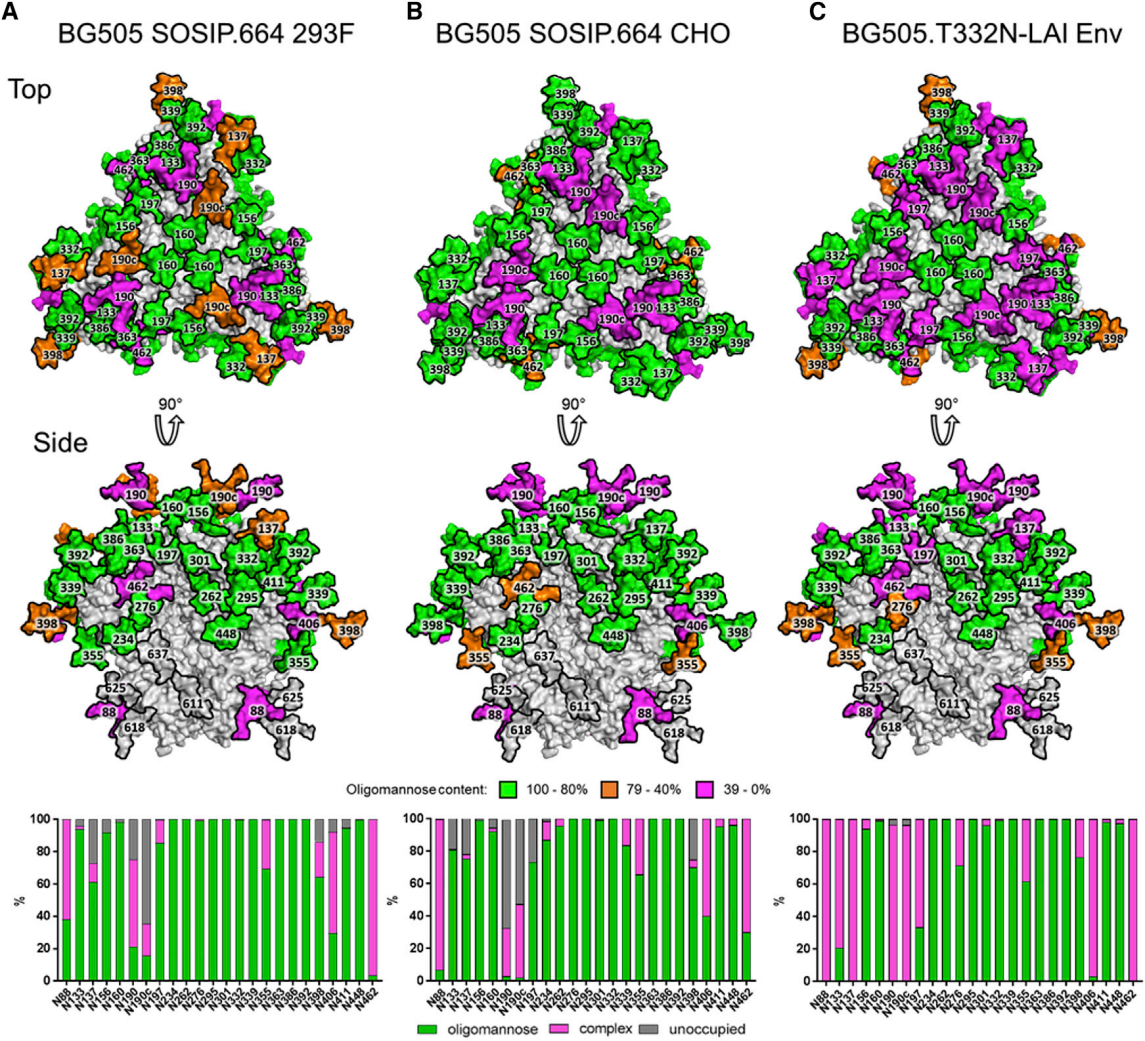
Determination of the Predominant Glycoforms Presented at Each Site on Soluble BG505 SOSIP.664 gp120 Expressed in Different Cells and Virus (A–C) A model of the predominant glycoform from 293F-derived BG505 SOSIP.664 using the cryo-EM model of BG505 SOSIP.664 (Behrens et al., 2016) is colored according to the predominant glycan species determined by glycosidase-treated glycopeptides for 293F cells (A), CHO cells (B), and BG505.T332N-LAI virus (C). The bar graphs represent the relative amounts of digested glycopeptides possessing the footprints for oligomannose glycans (green), complex-type glycans (magenta), and unoccupied PNGs (gray) at each gp120 site, listed from N to C terminus. See also Figure S1.

Using the above method, we were able to quantify the oligomannose-type glycans, complex-type glycans, and unoccupied potential N-glycosylation (PNG) sites across all 24 sites on the virion gp120. However, we could not isolate sufficient gp41 for a similar analysis. For comparison, we repeated the analysis using the recombinant BG505 SOSIP.664 trimers produced in 293F and CHO cells. The site-specific analysis reproduces the differences observed for the total glycoform analysis by UPLC; selected sites on gp120s from the BG505.T332N-LAI virus are more processed than on both the 293F-cell and the CHO-cell derived SOSIP.664 trimers. Elevated processing was not universal, but it was localized toward the apex of the trimer at sites such as N133, N137, and N197 (Figure 3). Hence, the previously described mannose patches IMP and TAMP are features shared not only by the two batches of recombinant SOSIP.664 trimer (293F and CHO) but also by the lymphoid cell-derived virion Env (Behrens and Crispin, 2017, Pritchard et al., 2015c). The N332-glycan is a “supersite of vulnerability” in that it is a key component of multiple bnAb epitopes (Kong et al., 2013, Pejchal et al., 2011). Because this glycan is consistently in an oligomannose form, the recombinant trimers correctly present the glycan components of the mannose patch bnAb epitopes. The processing of the TAMP was also conserved across the three trimer preparations. Thus, sites such as N160 and N156, which present complex-type glycan moieties when BG505 gp120 is expressed as a monomer (Behrens et al., 2017), are predominantly in oligomannose form on the virion-derived trimers and their recombinant SOSIP.664 comparators. This finding re-emphasizes the important influence of trimer formation on Env glycosylation and shows that it applies not only to recombinant trimers but also to virion-derived proteins (Behrens and Crispin, 2017, Behrens et al., 2016, Bonomelli et al., 2011, Doores et al., 2010, Pritchard et al., 2015a).

One notable difference was that the occupancy of glycan sites at certain regions across gp120 was lower for the soluble, truncated BG505 SOSIP.664 trimers than for their membrane-associated, full-length, virion-derived counterparts. This outcome was most noticeable around the V1/V2 region, where the respective percentage occupancy of N-glycosylation sites for 293F and CHO cell-derived trimers were N137 (73% and 78%), N190 (75% and 33%) and N190c (36% and 48%) (Figures 3A and 3B). In contrast, the same glycan sites on the BG505.T332N-LAI virion gp120 were >95% occupied (Figure 3C). The modestly under-glycosylated N137 site in V1 contributes to the epitope for PGT121 and related bnAbs, but the N190 and N190c sites in V2 play no such known role. Given their proximity to the trimer apex, the absence of glycans at these V2 sites may actually increase accessibility to various bnAb epitopes located in that region. However, partial occupancy has the effect of generating holes in the glycan shield on a subpopulation of the trimers, and such holes may be immunogenic (Crooks et al., 2015). In the case of glycan holes in the highly variable V1 and V2 regions, the likely outcome would be very-narrow-specificity nAbs or non-nAbs. It may become important to consider manufacturing strategies to modulate glycan site occupancy. We postulate that the codon-optimization process that is used to increase the production of recombinant proteins may result in faster translation and early folding events during Env biosynthesis that may reduce the likelihood of an N-glycan being attached; such an effect may be particularly apparent in the V1/V2 region, where several glycan sites are densely packed.

### Divergence and Conservation of Fine Processing at Various gp120 Glycan Sites

**Figure 4:**
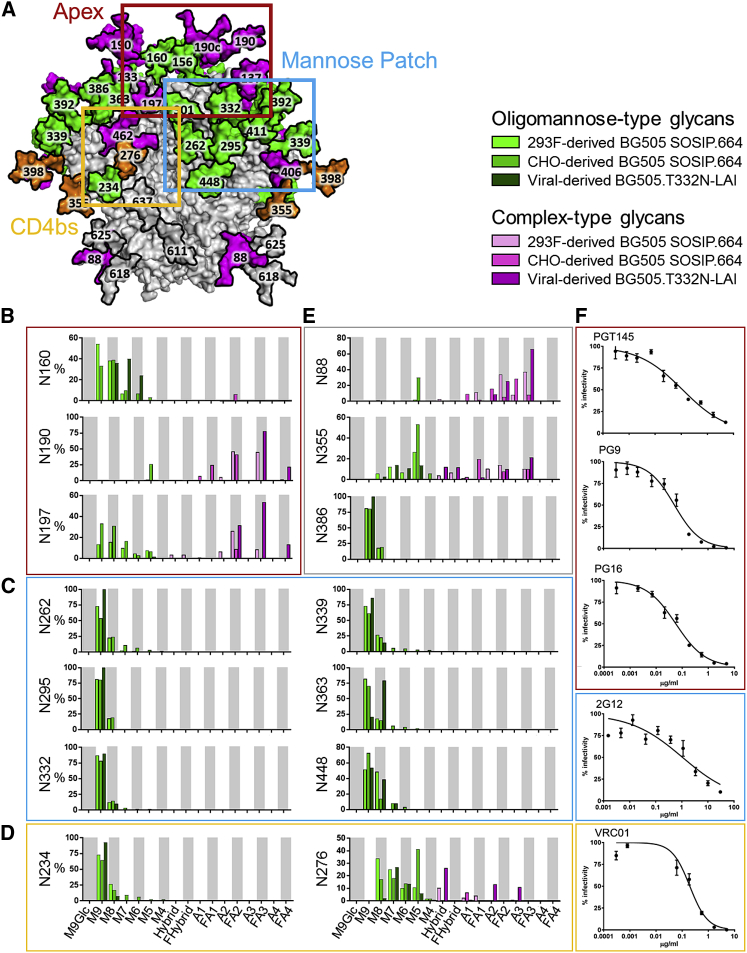
Compositional Site-Specific Analysis of Intact Glycopeptides from BG505 SOSIP.664 Trimers Produced in 293F or CHO Cells and from BG505.T332N-LAI Viruses (A) Model of the N-glycan compositions of BG505.T332N-LAI Env highlighting three regions that are important for bnAb binding (reproduced from Figure 3C). (B–E) Site-specific compositions of N-linked glycans located on (B) the trimer apex, (C) the mannose patch, (D) proximal to the CD4 binding site (CD4bs), and (E) at remaining sites on the trimer. (F) Neutralization by bnAbs targeting the apex (red), mannose patch (blue), and CD4bs (orange). Data corresponding to CHO-cell derived BG505 SOSIP.664 trimers are reproduced from Dey et al. (2018) and included here to assist in comparisons with other datasets. To simplify data presentation, complex glycans are grouped according to the presence or absence of fucose and the number of terminal antenna (e.g., FA2). Data are represented as means ± SEMs. See also Figure S1.

To investigate whether trimer immunogens correctly present key bnAb epitopes at a site-specific level, we characterized the glycans present on specific sites on the virion gp120 subunits (Figure 4). We used a previously validated glycopeptide-based liquid chromatography (LC)-MS approach (Behrens et al., 2016) to analyze the same batch of material that was used to prepare glycosidase-digested peptides. The inherent heterogeneity of N-linked glycosylation results in a diverse range of glycopeptides in the LC-MS analysis. We compared the new glycopeptide MS data for the BG505.T332N-LAI virion and 293F cell-derived BG505 SOSIP.664 trimers with our previously published information on the corresponding CHO cell-derived, CGMP-quality trimers (Dey et al., 2018). The commonality of the procedures used justifies this cross-study comparison.

A major class of bnAbs is directed against oligomannose-type glycans across the outer domain of gp120. Comparing the fine-processing states for N-linked glycan sites recognized by such bnAbs on the virion-derived trimer with the two versions of the SOSIP.664 trimer reveals the overall integrity of oligomannose processing; Man_9_GlcNAc_2_ dominates at the majority of IMP sites (Figure 4C). This finding applies to N332 and N339, which are critical to the 2G12 bnAb epitope, and is therefore consistent with the ability of 2G12 to neutralize the BG505.T332N-LAI virus (Figure 4F). The only notable deviation in the fine processing of oligomannose-type glycans was seen at N363, where Man_8_GlcNAc_2_ predominated on virion-derived trimers, but Man_9_GlcNAc_2_ did so on both of the recombinant trimer preparations.

Another region relevant to immunogen design strategies is the trimer apex, which contains highly conserved glycans, such as N160, that are involved in multiple bnAb epitopes, including PGT145, PG9, and PG16. Compared to gp120 monomers, the apex region of 293F cell-derived SOSIP.664 trimers is enriched for oligomannose-type glycans (Behrens et al., 2017). We assessed whether the same influence of trimerization on glycosylation applied to BG505.T332N-LAI virion-derived Env. The oligomannosylation seen at N160 (Figure 4B) is a strong indicator that the same steric restrictions do in fact apply. The high oligomannose content of the apex is also consistent with the ability of the PGT145, PG9, and PG16 bnAbs to neutralize the BG505.T332N-LAI virus (Figure 4F), because N160 mannose moieties are key components of their epitopes (Lee et al., 2017, Pancera et al., 2013).

While the trimer-specific restriction in glycan processing at the conserved N160 site applies to the BG505.T332N-LAI-derived Env and both preparations of BG505 SOSIP.664 trimers, we found differences at other sites around the apex. Thus, apex sites on the SOSIP.664 trimer that present a mixed population of oligomannose-type and complex-type glycans (e.g., N197) are more highly processed on the BG505.T332N-LAI virus (Figure 4B). In addition to elevated oligomannose processing, there was an increase in branching at the N197 site and at N190 (Figure 4B). The increased processing and branching are consistent with observations from the UPLC analyses (Figure 1) and are likely to contribute to the ∼10% increase in complex-type glycans on the BG505.T332N-LAI virus compared to the SOSIP.664 trimers.

The CD4 binding site (CD4bs) region also contains multiple bnAb epitopes, many of which are shielded or otherwise influenced by the highly conserved N276 glycan (Zhou et al., 2010). The N276 glycan was more extensively processed on the BG505.T332N-LAI virion Env than on either SOSIP.664 trimer (Figure 4D). Thus, the N276 site, together with N197, N190, and N462, constitute multiple sources of glycan heterogeneity surrounding the CD4bs. The heterogeneity observed here on the virion-derived material does not generate any VRC01-resistant virions (Figure 4F), presumably because the conformational plasticity of glycans means that even large glycans do not preclude VRC01 binding.

The BG505.T332N-LAI virus-derived material contained greater amounts of hybrid-type glycans than are present on both BG505 SOSIP.664 trimer preparations (Figure 2A). This finding was specifically the case for the N276 and N355 glycans (Figures 4D and 4E). There is growing interest in hybrid-type glycans because bnAbs such as PG16 preferentially recognize such structures (Shivatare et al., 2018). The hybrid glycan populations we have identified here do not correspond to known bnAb epitopes, but it will be important to seek out and characterize such structures in future studies of various Env proteins.

The N88 site near the base of the trimer is exclusively of the oligomannose type on gp120 from BaL virions (Panico et al., 2016). This glycan is structurally proximal to the membrane, and hence it is plausible that its processing may be governed by the same factors that apply to the nearby gp41 glycans. We found that the N88 glycan was exclusively complex on BG505.T332N-LAI gp120, but significantly less processed on both SOSIP.664 trimer preparations (Figures 3 and 4E). These observations further illustrate how proximity to the plasma membrane may influence how glycan-processing enzymes act on certain sites, irrespective of the Env genotype.

### Perspectives

We have shown that both the global and the site-specific glycosylation of virion-derived Env is largely recapitulated by recombinant SOSIP.664 trimers expressed either transiently in 293F cells or stably in CHO cells under CGMP conditions. These observations are consistent with the finding that the recombinant trimers display the epitopes for numerous glycan-targeting bnAbs that neutralize the corresponding virus and support their exploration as clinical immunogens (Dey et al., 2018, Sanders et al., 2013). However, we also detected notable points of divergence between the viral and recombinant proteins that may help define how best to produce Env protein immunogens. In particular, the trimers from both 293F and CHO cells display reduced levels of glycan branching and terminal processing than their virion-derived counterparts. This trend occurs both at sites that are always fully complex type and at mixed sites where the levels of oligomannose-type glycans are reduced on virus-derived Env. As a result of the difficulties of eliciting glycan-binding bnAbs, it may be advisable to avoid exposing the immune system to glycan epitopes that are not presented on viral Env. It will be important to extend these types of glycan analysis to viruses and SOSIP trimers of additional genotypes, with the goal of refining how trimer-based immunogens are glycosylated. It will also be critical to understand the site-specific glycosylation of virus from PBMCs or primary T cells because we can expect to discover cell-dependent influences on glycosylation patterns. The next generation of clinical immunogens may also benefit from the development of strategies to boost the immunogenicity of glycan-dependent epitopes.

## STAR★Methods

### Key Resources Table

**Table:** 

REAGENT or RESOURCE	SOURCE	IDENTIFIER
**Antibodies**		

VRC01	This paper	RRID: AB_2491019
2G12	This paper	N/A
PG9	This paper	RRID: AB_2491030
PG16	This paper	RRID: AB_2493031
PGT145	This paper	RRID: AB_2491054
PGT151	This paper	N/A

**Cell Lines**		

HEK293T cells	ATCC	Cat# 11268
A66-R5	From Dr. James Hoxie, University of Pennsylvania	N/A
HEK293F cells	Thermo Fisher Scientific	Cat# R79007

**Virus Strains**		

SIVmac239/SupT1-R5	AIDS and Cancer Virus Program, NCI-Frederick	N/A
HIV-1 BAL/SupT1-R5	AIDS and Cancer Virus Program, NCI-Frederick	N/A
HIV-1 NL4-3/SupT1	AIDS and Cancer Virus Program, NCI-Frederick	N/A

**Chemicals, Peptides, and Recombinant Proteins**		

10% fetal bovine serum	Gemini GemCell	Lot# A90D69E
FreeStyle MAX Reagent	Thermo Fisher Scientific	Cat# 10259172
GIBCO OptiPRO SFM	Thermo Fisher Scientific	Cat# 10569520
FreeStyle 293F media	Thermo Fisher Scientific	Cat# 12338026
Acetonitrile, 80%, 20% Water with 0.1% Formic Acid, Optima LC/MS	Fisher Scientific	Cat# 15431423
Water with 0.1% Formic Acid (v/v), Optima LC/MS Grade	Fisher Scientific	Cat# LS118-212
RPMI 1640	Thermo Fisher Scientific	Cat# 11875093
TransIT-293	Muris	Cat# MIR 2700
SYPRO Pro-Q Emerald	Molecular Probes	Cat# P33378
SYPRO Ruby	Molecular Probes	Cat# S12000
BigDye Terminator	Applied Biosystems	Cat# 4337454
L-glutamine	Sigma-Aldrich	Cat# G3126
acetonitrile	Fisher Scientific	Cat# 10489553
trifluoroacetic acid	Fisher Scientific	Cat# 10155347
procainamide hydrochloride	Abcam	Cat# ab120955
H_2_O^18^	Sigma-Aldrich	Cat# 329878
dithiothreitol	Sigma-Aldrich	Cat# 43819
iodacetamide	Sigma-Aldrich	Cat# I1149
ammonium formate buffer	Waters	Cat# 186007081
sodium cyanoborohydride	Sigma-Aldrich	Cat# 156159
DMSO	Sigma-Aldrich	Cat# D2438
acetic acid	Fisher Scientific	Cat# 10384970
rHIV-1 ADA gp120	From Drs. Bridget Puffer and Robert Doms, University of Pennsylvania	N/A
HIV-1 MN p24 purified from HIV-1 MN	AIDS and Cancer Virus Program, NCI-Frederick	N/A
PNGase F	New England BioLabs	Cat# P0705S
Endoglycosidase H	New England BioLabs	Cat# P0702S
α2-3, 6, 8 neuraminidase	New England BioLabs	Cat# P0720S
α2-3 neuraminidase	New England BioLabs	Cat# P0743S
mass spectrometry grade trypsin	Promega	Cat# V5280
sequencing grade chymotrypsin	Promega	Cat# V1061

**Critical Commercial Assays**		

p24 antigen capture immunoassay	AIDS and Cancer Virus Program	N/A

**Oligonucleotides**		

Primers for Viral Sequencing EnvB5out 5′ TAG AGC CCT GGA AGC ATC CAG GAAG-3′	This paper	N/A
Primers for Viral Sequencing EnvB3out 5′-TTG CTA CTT GTG ATT GCT CCA TGT-3′	This paper	N/A
Primers for Viral Sequencing EnvB5in 5′-CAC CTT AGG CAT CTC CTA TGG CAG GAA GAAG-3′	This paper	N/A
Primers for Viral Sequencing EnvB3in 5′-GTC TCG AGA TAC TGC TCC CAC CC-3′	This paper	N/A

**Recombinant DNA**		

BG505.T332N-LAI IMC	This paper	N/A
BG505 SOSIP.664	This paper	N/A

**Software and Algorithms**		

VersaDoc 3000 Imaging Software	Bio-Rad Laboratories	N/A
Empower 3.0	Waters	N/A
Masslynx v4.1	Waters	N/A
Driftscope version 2.8	Waters	N/A
ByonicTM (Version 2.7)	Protein Metrics	N/A
ByologicTM software (Version 2.3)	Protein Metrics	N/A
GraphPad Prism (Version 7)	GraphPad Software	N/A

**Other**		

5.0 μm Millipore Opticap XL 10 capsule filter	Millipore	CAT# KN50A10HH1
0.5 μm Millipore Opticap XL 5 capsule filter	Millipore	CAT# K005A05HH1
Poros R2/H column (2.1 × 100 mm)	Applied Biosystems	Cat# 1111416
QIAGEN column (QIAamp DNA blood kit)	QIAGEN	Cat# 51104
High Fidelity Platinum Taq	Thermo Fisher Scientific	Cat# 11304011
HisTrap HP column	GE Healthcare	Cat# 17-5248-02
Spe-ed Amide 2 cartridges	Applied Separations	Cat# 4821
Glycan BEH Amide column (2.1 mm x 100 mm, 1.7 μM)	Waters	Cat# 186004741
PVDF protein-binding membrane	Millipore	Cat# MAIPS4510
Vivaspin 500, 3 kDa MWCO Polyethersulfone	Sigma	Cat# GE28-9322-18
SDS-PAGE 4-20% Tris-glycine gel	Invitrogen	Cat# XP04205BOX
C18 ZipTip	Merck Milipore	Cat# ZTC18S008
EasySpray PepMap RSLC C18 column (75 μm x 75 cm)	Thermo Fisher Scientific	Cat# ES805
Nafion perfluorinated membrane	Sigma-Aldrich	Cat# 274674-1EA

### Contact for Reagent and Resource Sharing

Further information and requests for resources and reagents should be directed to and will be fulfilled by the Lead Contact; Max Crispin (Max.Crispin@soton.ac.uk)

### Experimental Model and Subject Details

#### HEK293T cell culture

An infectious virus stock was prepared by transfecting 293T cells (American Type Culture Collection Cat. #11268) with the IMC using the TransIT®-293 transfection reagent as described by the manufacturer (Muris Inc).

#### A66-R5 T cell line

The A66-R5 cell line (a gift from Dr. James Hoxie, University of Pennsylvania, Philadelphia, PA) is a derivative of the SUP-T1 cell line (Smith et al., 1988) that expresses CCR5 but not CXCR4 (Del Prete et al., 2009). The culture was maintained in RPMI 1640 containing L-glutamine and supplemented with 10% fetal bovine serum (Gemini GemCell Lot A90D69E, heat inactivated) and antibiotics (1000 U/mL penicillin/1000 μg/mL streptomycin).

#### HEK293F cell culture and transfection

HEK293F cells were maintained at a density of 1-3x10^6^ cells per mL at 37 degrees and 125rpm shaking. A plasmid encoding BG505 SOSIP.664 containing a C-terminal His-tag was transiently co-transfected with a Furin-encoding plasmid (4:1) in HEK293F cells. The cells were transfected at a density of 1x10^6^ cells per ml and incubated for 5 days at 37 degrees with 8% CO_2_ and 125rpm shaking.

### Method Details

#### Production of BG505.T332N-LAI gp120 and gp41

An infectious stock of HIV-1 BG505.T332N-LAI was derived by transfecting 293T cells with the infectious molecular clone (IMC). The IMC Env glycoproteins have gp120 and gp41-ectodomain subunits derived from the primary R5 Tier-2, clade A BG505 week 6 isolate, but with the N332-glycan knocked-in to mimic the BG505 SOSIP.664 soluble trimer sequence (Medina-Ramírez et al., 2017, Sanders et al., 2013). The transfection stock was used to infect the CD4+, CCR5+ A66-R5 T cell line to provide the large amount of virus required for glycan analysis. Cytopathic effects were first observed in the culture on day-18 post infection, and progeny virus was first detected by p24 antigen capture immunoassay on day-21. Because the IMC was cytopathic, ∼100-500,000 uninfected A66-R5 cells were added per mL of the culture at seven different time points during its 54-day duration, to maintain a high level of viability and sustain virus production. On day-54, 20 L of culture supernatant were harvested for virion purification by sucrose density-gradient centrifugation. The resulting 1,000-fold concentrated stock (lot P4408, 20 mL volume) of HIV-1 BG505.T332N-LAI/A66-R5 virus served as the starting material for purifying gp120 and gp41 Env glycoproteins. The mass amounts of p24 Gag protein and gp120 Env glycoprotein in the purified virus stock were estimated by analyzing fluorescent dye-stained SDS-PAGE gel bands using a sensitive, densitometric technique, compared to standard curves derived using highly purified viral protein preparations; well characterized reference virus preparations (SIVmac239/SupT1-R5, HIV-1 BAL/SupT1-R5 and HIV-1 NL4-3/SupT1) served as additional controls.

##### Cell culture production of BG505.T332N-LAI virus

The construction of the BG505.T332N-LAI IMC has been described previously (Medina-Ramírez et al., 2017). Neutralization assays using this virus were performed at the Academic Medical Centre, Amsterdam as previously described (Sanders et al., 2015). An infectious virus stock was prepared by transfecting 293T cells (American Type Culture Collection Cat. #11268) with the IMC using the TransIT®-293 transfection reagent as described by the manufacturer (Muris Inc). Aliquots of cell free culture supernatant (infectious stock) were collected and stored at −80°C. To produce the BG505.T332N-LAI virus, a 50-mL starter culture of A66-R5 cells containing 500,000 viable cells/mL was inoculated with 1 mL of the above infectious stock. The culture was maintained in RPMI 1640 containing L-glutamine and supplemented with 10% fetal bovine serum (Gemini GemCell Lot A90D69E, heat inactivated) and antibiotics (1000 U/mL penicillin/1000 μg/mL streptomycin). Depending upon the cell density, the cultures were passaged at 1:2 to 1:5, twice weekly during scale up. Progeny virus production was monitored using an in-house HIV-1 p24 antigen capture immunoassay (AIDS and Cancer Virus Program, Frederick National Laboratory for Cancer Research).

##### Purification of BG505.T332N-LAI virus

Supernatant from the infected A66-R5 cell culture was sequentially filtered to remove cells and large extracellular vesicles using 5.0 μm (Millipore Opticap® XL 10) and 0.5 μm (Millipore Opticap® XL 5) capsule filters, respectively. The viruses present were purified by continuous flow sucrose density gradient centrifugation followed by sucrose removal, and the final virus concentration was determined, all via procedures described elsewhere (Chertova et al., 2002). Aliquots of the purified virus (lot P4408) were stored in a liquid N_2_ vapor phase freezer prior to use.

##### Reversed-phase HPLC purification of virion gp120 and HPLC plus SDS-PAGE purification of virion gp120 and gp41

The virus preparation (lot P4408) described above was disrupted in 8M Guanidine-HCl (Pierce, Rockford, IL) and the proteins fractionated by HPLC under non-reducing conditions to isolate the gp120 and gp41 components. HPLC was performed at a flow rate of 300 μL/min on a 2.1 × 100 mm Poros® R2/H narrow bore column (Applied Biosystems, Bedford, MA, USA), using aqueous acetonitrile/trifluoroacetic acid solvents and a Shimadzu HPLC system equipped with LC-10AD pumps, SCL-10A system controller, CTO-10AC oven, FRC-10A fraction collector and SPD-M10AV diode array detector. The gradient of buffer B (0.1% trifluoracetic acid in acetonitrile) was: 20%−36%, 5 min; 36%−43%, 14 min; 43%–50%, 11 min; 50%–80%, 5 min; and 80%, 5 min. A temperature of 55°C was maintained during HPLC separation. Protein peaks were detected by UV absorption at 206 and 280 nm. Fractions corresponding to gp120 and gp41 were subjected to SDS-PAGE. The gel bands corresponding to gp120 and gp41 were excised and stored at −20°C for use in UPLC and mass spectrometry analyses of released glycans.

A second aliquot of virus was processed by HPLC in an identical manner, resulting in the purification of ∼70 μg of gp120 subunits, as quantified using the fluorescent dye staining technique. Fractions containing the gp120 protein were collected, pooled and lyophilized. This purified gp120 stock was used for subsequent site-specific glycan analysis.

##### Sypro dual color fluorescent protein gel analysis

Proteins from lysed virus preparations were resolved by SDS-PAGE on 4%–20% Tris-glycine gels (Invitrogen) under reducing conditions. The p24-Gag and gp120-Env contents of the samples were determined by a two-color fluorescence staining assay. Gels with virus samples and a dilution series of purified protein standards were stained with two fluorescent dyes (Molecular Probes, Eugene, OR), SYPRO Pro-Q Emerald (green fluorescence) to detect glycoproteins, such as gp120, and SYPRO Ruby (red fluorescence) to detect all proteins, including p24. Stained gels were analyzed for fluorescence at 520 nm with UV excitation, using a VersaDoc 3000 Imaging System (Bio-Rad Laboratories, Hercules CA) software package (Bio-Rad Laboratories). The integrated pixel density signals for the p24 and gp120 bands from the unknown samples were interpolated onto a standard curve derived by a linear regression analysis of optical density values corresponding to serial dilutions of reference standard proteins. The latter were highly purified, amino acid composition-quantified recombinant vaccinia-produced HIV-1 ADA gp120 (provided by Drs. Bridget Puffer and Robert Doms, University of Pennsylvania, Philadelphia, PA) or HIV-1 MN virion-derived p24 (AIDS and Cancer Virus Program, NCI-Frederick, Frederick, MD). Well-characterized reference preparations of SIVmac239/SupT1-R5, HIV-1 BAL/SupT1-R5 and HIV-1 NL4-3/SupT1 viruses (AIDS and Cancer Virus Program, NCI-Frederick, Frederick, MD) were used to validate this procedure (Del Prete et al., 2009, Louder et al., 2005). The amount of gp41 that was purified is assumed to be comparable to gp120, but it was not measured directly.

#### Viral genome sequencing

Genomic DNA was isolated from the HIV-1 BG505.T332N-LAI infected A6R5 cells on day 54 of the culture using a QIAGEN column (QIAamp DNA blood kit). DNA was then serially diluted and the entire *env* gene was PCR-amplified using High Fidelity Platinum Taq (Thermo Fisher Scientific) with 1x buffer, 2 mM MgCl_2_, 0.2 mM of each dNTP, and 0.025 U/μL Taq polymerase and the appropriate primers at a concentration of 0.2 μM. The primers for the first-round PCR were EnvB5out 5′ TAG AGC CCT GGA AGC ATC CAG GAAG-3′ and EnvB3out 5′-TTG CTA CTT GTG ATT GCT CCA TGT-3′. The primers for the second-round PCR were EnvB5in 5′-CAC CTT AGG CAT CTC CTA TGG CAG GAA GAAG-3′ and EnvB3in 5′-GTC TCG AGA TAC TGC TCC CAC CC-3′. The cycler conditions for the first round PCR were 94°C for 2 min, followed by 35 cycles of 94°C for 15 s, 55°C for 30 s, and 68°C for 4 min, followed by a final extension of 68°C for 10 min. The product of the first-round PCR (1 μL) was subsequently used as a template in a second-round of PCR using the same conditions but for a total of 45 cycles. All PCR-positive amplicons were directly sequenced using BigDye Terminator chemistry (Applied Biosystems). Any sequence with evidence of double peaks was excluded from further analysis. *Env* sequences were compared to the reference HIV-1 BG505.T332N-LAI IMC. Only one polymorphism from the reported IMC sequence was seen consistently; a single nucleotide change created a point substitution, G314R, at the tip of the gp120 V3-region: GP**G**Q to GP**R**Q. This very rare sequence change is found in only 2 of ∼5500 V3 sequences in the Los Alamos Sequence Database. Its functional significance, if any, is not known. While a few other changes were seen in the swarm, none was present in more than one of the five sequences obtained. The knocked-in T332N substitution was consistently retained.

##### Production of BG505 SOSIP.664 trimers

Correctly folded, cleaved BG505 SOSIP.664 trimers were purified from the supernatants using a Ni^2+^-NTA affinity column (GE Healthcare UK) followed by bnAb PGT151 affinity chromatography, as described previously (Ringe et al., 2015). The production and purification of the same BG505 SOSIP.664 trimers from a stable CHO cell line under cGMP conditions has been described elsewhere (Dey et al., 2018).

##### In-gel digestion of N-glycans

The SDS-PAGE gel bands corresponding to gp120 and gp41 were excised and washed with 100% acetonitrile and water three times. The N-glycans for UPLC and ion mobility mass spectrometry analyses were then enzymatically released by within-gel digestion by PNGase F (New England BioLabs) for 16 h at 37°C.

##### UPLC N-glycan analysis

Aliquots of released glycans were fluorescently labeled with procainamide using a prepared labeling solution (110 mg/mL procainamide, 60 mg/mL sodium cyanoborohydride in 30% DMSO, 70% acetic acid) at 65°C for 4 hours. Excess label and PNGaseF was removed using Spe-ed Amide 2 cartridges (Applied Separations). Glycans were analyzed on a Waters Acquity H-Class UPLC instrument with a Glycan BEH Amide column (2.1 mm x 100 mm, 1.7 μM, Waters) and the following gradient: time (t) = 0: 22% A, 78% B (flow rate = 0.5 mL/min); t = 38.5: 44.1% A, 55.9% B (0.5 mL/min); t = 39.5: 100% A, 0% B (0.25 mL/min); t = 44.5: 100% A, 0% B (0.25 mL/min); t = 46.5: 22% A, 78% B (0.5 mL/min), where solvent A was 50 mM ammonium formate (pH 4.4) and B was acetonitrile. Fluorescence was measured at an excitation wavelength of 310 nm and an emission wavelength of 370 nm. Data were processed using Empower 3 software (Waters, Manchester, UK). The relative abundance of N-glycan structures was determined by digesting fluorescently labeled glycans with the following glycosidases (New England Biolabs) at 37°C for 16 h: Endoglycosidase H (endoH), α2-3, 6, 8 neuraminidase and α2-3 neuraminidases. Glycans were extracted using a PVDF protein-binding membrane (Millipore) and analyzed as described above.

##### N-glycan mass spectrometry

Immediately prior to MS analysis, a separate unlabeled aliquot of glycans were further desalted for 30 min on top of a Nafion membrane, as described elsewhere (Börnsen et al., 1995), and a trace amount of ammonium phosphate was added to promote phosphate adduct formation. Glycans were analyzed by nano-electrospray (ESI) with direct infusion using a Synapt G2Si instrument (Waters, Manchester, UK) with the following settings: capillary voltage, 0.8-1.0 kV; sample cone, 150 V; extraction cone, 150 V; cone gas, 40 l/h; source temperature, 80°C; trap collision voltage, 4-160 V; transfer collision voltage, 4 V; trap DC bias, 60 V; IMS wave velocity, 450 m/s; IMS wave height, 40 V; trap gas flow, 2 ml/min; IMS gas flow, 80 ml/min. Data were acquired and processed with MassLynx v4.1 and Driftscope version 2.8 software (Waters, Manchester, UK). Structural assignments were based on the identification of cross-ring and D-type fragments that are characteristic for negatively charged glycan ions (Harvey, 2005). The nomenclature used to describe the fragment ions is that devised by Domon and Costello (Domon and Costello, 1988).

##### Glycopeptide mass spectrometry

Env proteins were denatured, reduced, and alkylated by sequential 1 h incubations at room temperature (RT) in the following solutions: 50 mM Tris/HCl, pH 8.0 buffer containing 6 M urea and 5 mM dithiothreitol (DTT), followed by the addition of 20 mM iodacetamide (IAA) for a further 1h at RT in the dark, and then additional DTT (20 mM), to eliminate any residual IAA. The alkylated trimers were buffer-exchanged into 50 mM Tris/HCl, pH 8.0 using Vivaspin columns and digested separately with trypsin and chymotrypsin (Mass Spectrometry Grade, Promega) at a ratio of 1:30 (w/w). Reaction mixtures were dried and glycopeptides were extracted using C18 Zip-tip (MerckMilipore) following the manufacturer’s protocol.

Eluted glycopeptides were dried again and re-suspended in 0.1% formic acid prior to mass spectrometry analysis. An aliquot of intact glycopeptides was analyzed by nanoLC-ESI MS with an Easy-nLC 1200 system coupled to an Orbitrap Fusion mass spectrometer (Thermo Fisher Scientific) using higher energy collisional dissociation (HCD) fragmentation as previously described (Behrens et al., 2016). Briefly, peptides were separated using an EasySpray PepMap RSLC C18 column (75 μm x 75 cm) with a 275 minute linear gradient consisting of 0%–32% acetonitrile in 0.1% formic acid over 240 minutes followed by 35 minutes of 80% acetronitrile in 0.1% formic acid. The flow rate was set to 200 nL/min. The spray voltage was set to 2.8 kV and the temperature of the heated capillary was set to 275°C. HCD collision energy was set to 50%, appropriate for fragmentation of glycopeptide ions. Glycopeptide fragmentation data were extracted from the raw file using Byonic™ (Version 2.7) and Byologic™ software (Version 2.3; Protein Metrics Inc.). The glycopeptide fragmentation data were evaluated manually for each glycopeptide; the peptide was scored as true-positive when the correct b and y fragment ions were observed along with oxonium ions corresponding to the glycan identified. The chromatographic areas for each true-positive peptide with the same amino acid sequence were compared to allow the relative amounts of each glycoform at each site to be determined.

##### Glycopeptide occupancy analysis

The remaining glycopeptides were first digested with endoH (New England Biolabs) to deplete oligomannose- and hybrid-type glycans and leave a single GlcNAc residue at the corresponding site. The reaction mixture was then dried completely and resuspended in a mixture containing 50 mM ammonium bicarbonate and PNGase F (New England Biolabs) using only H_2_O^18^ (Sigma-Aldrich) throughout. This second reaction cleaves the remaining complex-type glycans but leaves the GlcNAc residues remaining after endoH cleavage intact. The use of H_2_O^18^ in this reaction enables complex glycan sites to be differentiated from unoccupied glycan sites as the hydrolysis of the glycosidic bond by PNGaseF leaves a heavy oxygen (O^18^) isotope on the resulting aspartic acid residue. The resultant peptides were purified as outlined above and subjected to reverse-phase (RP) nanoLC-MS similar to the aforementioned glycopeptide analysis. Instead of the extensive N-glycan library used above, two modifications were searched for: +203 Da corresponding to a single GlcNAc, a residue of an oligomannose/hybrid glycan, and +3 Da corresponding to the O^18^ deamidation product of a complex glycan. A lower HCD energy of 27% was used as glycan fragmentation was not required. Data analysis was performed as above and the relative amounts of each glycan determined, including unoccupied peptides.

### Quantification and Statistical Analysis

The integration of peaks corresponding to fluorescently labeled N-glycans shown in Figure 1 was performed using Empower 3.0 (Waters). Neutralization data shown in Figure 4 was plotted and the mean and SEM was calculated using GraphPad Prism v7. Chromatographic areas were extracted for site-specific analysis, displayed in Figure 3 and 4, using Byonic™ (Version 2.7) and Byologic™ software (Version 2.3) by Protein Metrics.

## Supplemental Information

Document S1. Figures S1–S4 and Tables S1 and S2

Document S2. Article plus Supplemental Information
