# Supplementary material for: Site-Specific Glycosylation of Virion-Derived HIV-1 Env Is Mimicked by a Soluble Trimeric Immunogen
Source: Cell Rep. 2018 Aug 24;24(8):1958–1966.e5. doi: 10.1016/j.celrep.2018.07.080 (PMC6113929; doi:10.1016/j.celrep.2018.07.080)
Supplement: Document S1. Figures S1–S4 and Tables S1 and S2 [file mmc1.pdf]

Cell Reports, Volume *24*

Supplemental Information

Site-Specific Glycosylation of Virion-Derived HIV-1 Env Is Mimicked by a Soluble Trimeric Immunogen

Weston B. Struwe, Elena Chertova, Joel D. Allen, Gemma E. Seabright, Yasunori Watanabe, David J. Harvey, Max Medina-Ramirez, James D. Roser, Rodman Smith, David Westcott, Brandon F. Keele, Julian W. Bess Jr., Rogier W. Sanders, Jeffrey D. Lifson, John P. Moore, and Max Crispin

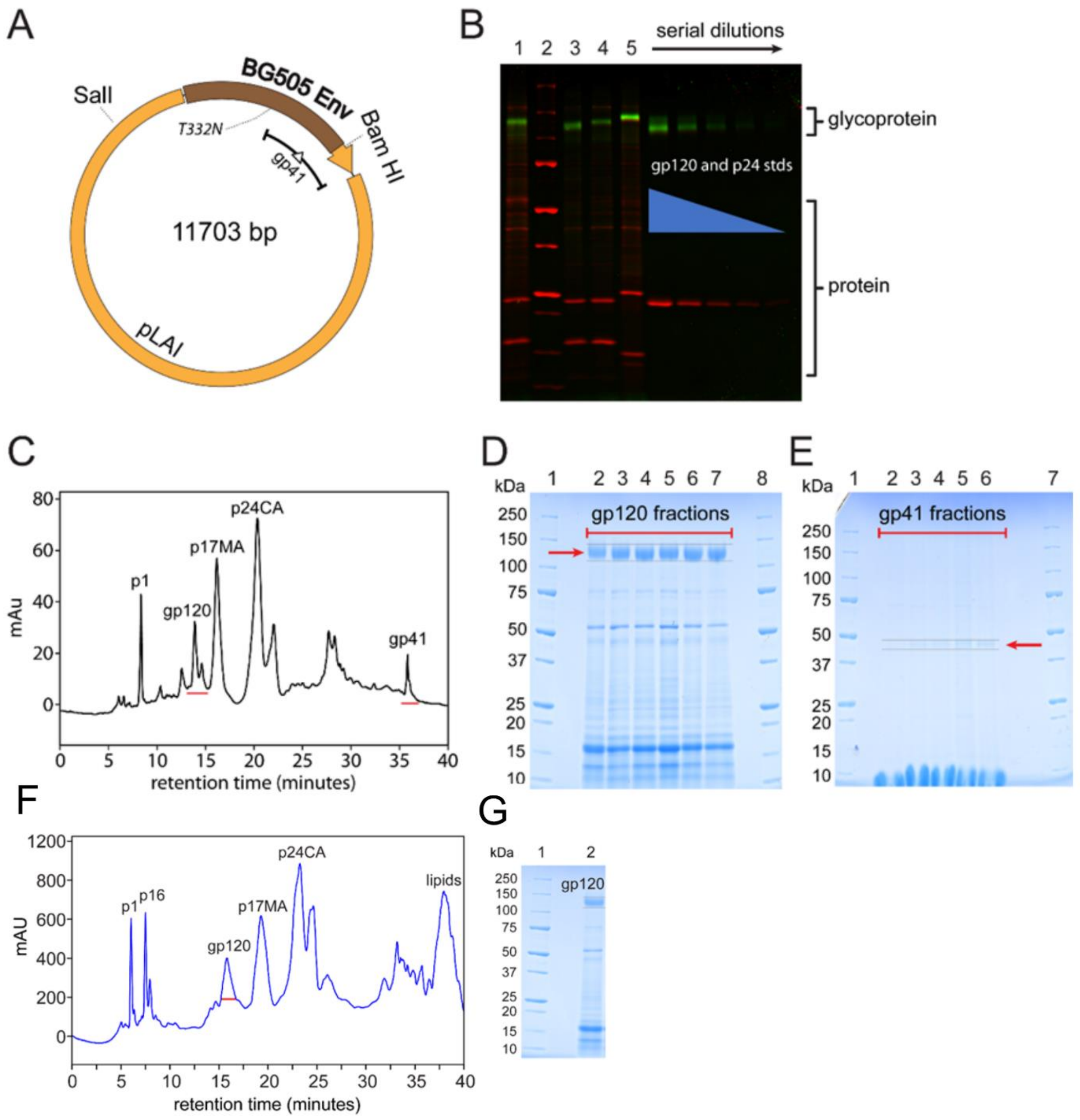

Figure S1. Construction and purification of the BG505.T332N-LAI infectious molecular clone (IMC) and its Env proteins. (A) Schematic representation of the BG505.T332N-LAI IMC. (B) Lane 1: Two-dye SYPRO-stained (total protein, red; glycoproteins, green) SDS-PAGE gel analysis of BG505.T332N-LAI. Lane 2: M.W. standards. Lanes 3–5: Positive control samples (3: HIV-1 BAL, 4: HIV-1 NL4/3, 5: SIVmac239). The gel-staining was calibrated using the gp120 (200–12.5 ng) and purified p24 (600–37.5 ng) standards shown on the right. (C) HPLC separation of BG505.T332N-LAI/A66-R5 (P4408) under non-reducing conditions. UV absorbance was measured at 206 nm. Viral protein peaks (identified by subsequent SDS-PAGE gel, protein sequencing and immunoblot analyses) are labelled above the chromatogram. (D) SDS-PAGE gel of gp120. Lanes 1 and 8: M.W. standards, Lane 2-7: pooled HPLC fractions underscored in red on panel C. (E) SDS-PAGE gel of gp41. Lanes 1 and 7: M.W. standards, Lanes 2-6: pooled HPLC fractions are underscored in red on panel C. The gel bands in D (gp120) and E (gp41) indicated with red arrows were excised and used for released glycan analysis by UPLC and mass spectrometry. (F) HPLC separation of BG505.T332N-LAI/A66-R5 (P4408) under non-reducing conditions for site-specific analysis. Viral protein peaks (identified by subsequent SDS-PAGE gel, protein sequencing and immunoblot analyses) are labelled above the chromatograph. (G) SDS-PAGE gel of gp120. Lane 1: M.W. standards, lane 2: pooled HPLC fractions underscored in red on panel A. This HPLC-purified material was subsequently used for site-specific glycan analysis. Related to Figure 1, Table S1, Figure 3 and Figure 4.

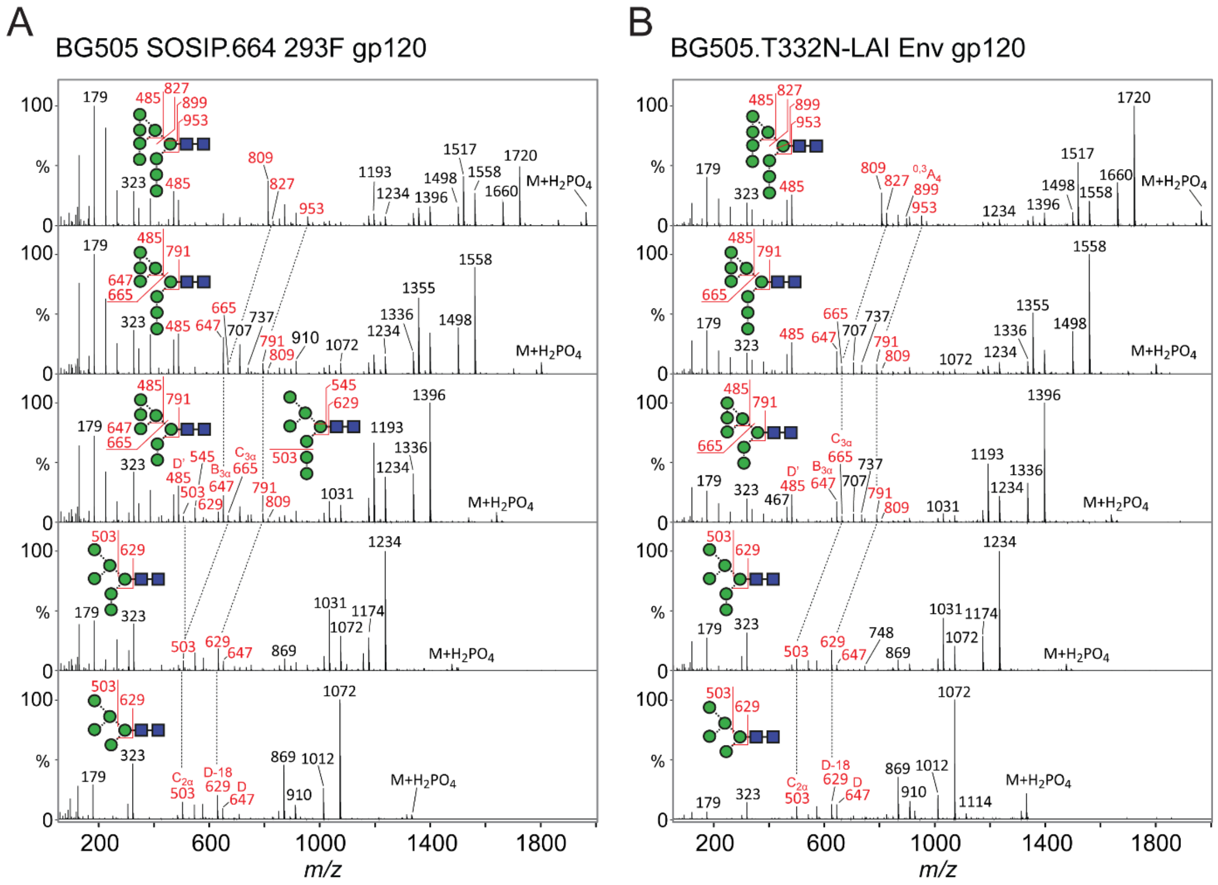

Figure S2: MS/MS fragmentation spectra of oligomannose-type glycans to determine their fine structure. (A) Fragmentation spectra of oligomannose-type glycans released from 293F-derived BG505 SOSIP.664 with diagnostic structural ions highlighted in orange. (B) Fragmentation spectra of oligomannose-type glycans released from 293F-derived BG505.T332N-LAI with diagnostic structural ions highlighted in orange. Peaks are annotated according to the scheme proposed by Domon and Costello (Domon and Costello, 1988). Related to Figure 2.

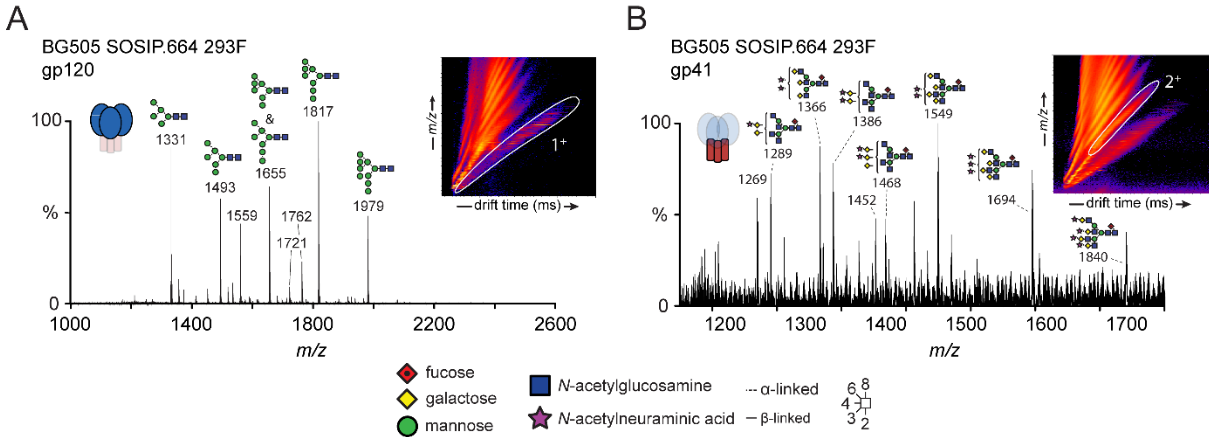

Figure S3: Ion mobility mass spectrometric analysis of gp120 and gp41 N-glycans released from BG505 SOSIP.664 produced in 293F cells. (A) Mobility-extracted singly charged negative ions found on BG505 SOSIP.664 gp120. The corresponding singly charged ions are encircled with the white oval in the DriftScope image (m/z against drift time). (B) Mobility-extracted doubly charged negative ions found on 293F-derived gp41. The corresponding doubly charged ions are encircled with the white oval in the DriftScope image (*m/z* against drift time). Related to Figure 2.

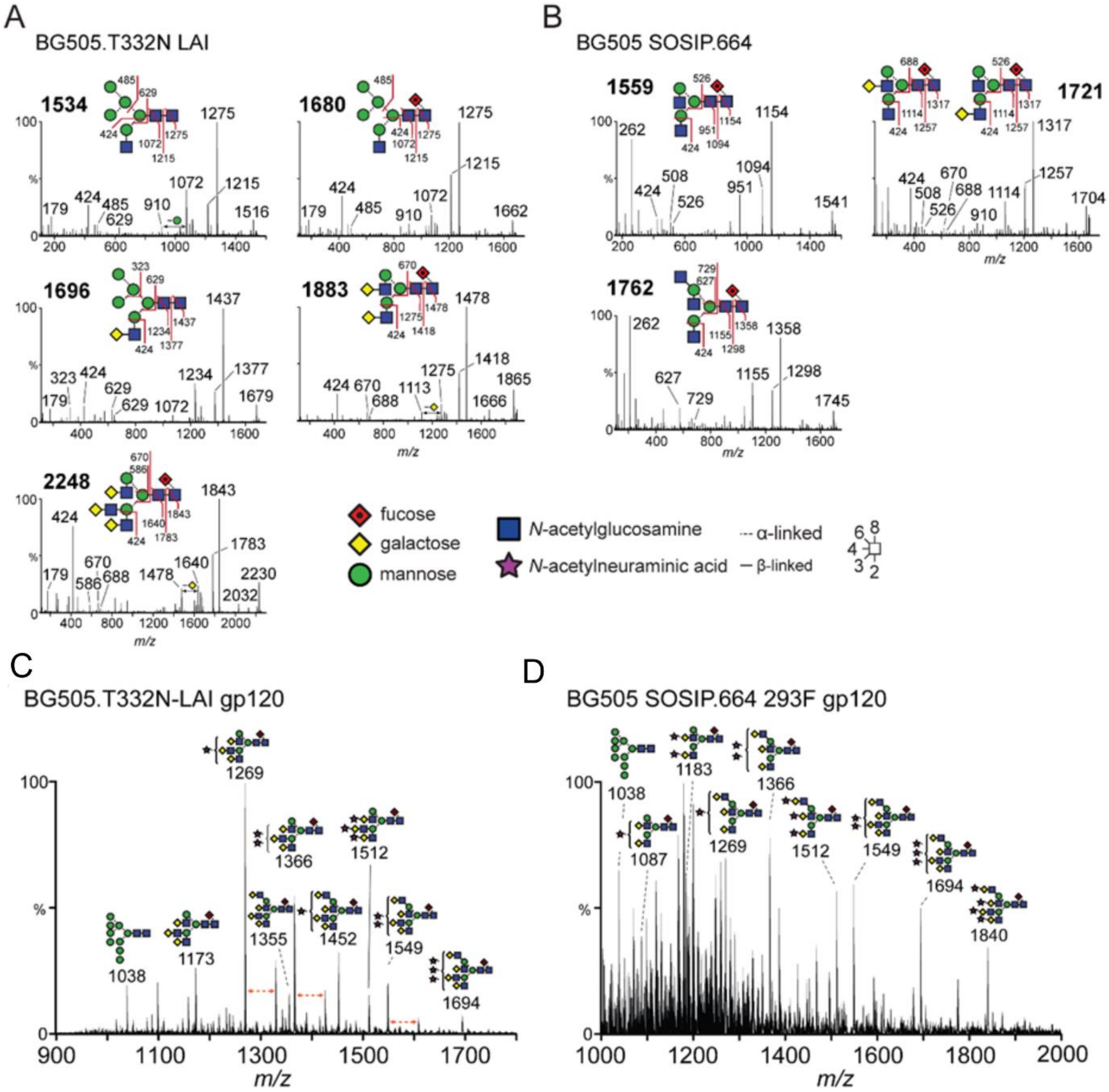

Figure S4: MS/MS fragmentation spectra of BG505.T332N-LAI gp120 and BG505 SOSIP.664 complex N-glycans. (A) MSMS fragmentation spectra to determine the fine structure of the top 5 most common singly charged ions corresponding to complex-type glycans released from BG505.T332N-LAI gp120. (B) MSMS fragmentation spectra to determine the fine structure of the top 3 most common singly charged ions corresponding to complex-type glycans released from BG505 SOSIP.664 gp120 produced in 293F cells. (C) Doubly charged N-glycans extracted in Driftscope based on their *m/z* and retention time through the ion mobility cell from gp120 derived from BG505.T332N-LAI virions. Peaks with additional phosphate adducts are labelled in orange. (D) Doubly charged N-glycans from gp120 derived from BG505 SOSIP.664 produced in 293F cells. Related to Figure 2.

sample	gag:env	~trimer
P4408 HIV-1 LAI BG505.T332N/A65-R5	24	20
P4239 HIV-1 BAL/SupT1-R5	16	28
P4249 HIV-1 NL43/SupT1	42	11
P4004 SIVmac 239/SupT1-R5	10	48

Table S1: Calculated Gag:Env ratios for the HIV-1 LAI BG505.T332N/A66-R5 P4408 preparation and reference standard virus preparations based on densitometric measurements, normalized for molecular weights. The average number of trimers per virion was estimated assuming there are 1400 Gag molecules present (Zhu et al., 2003). Related to Figure 1.

		gp120			gp41		
		Viral-derived	SOSIP 293F	SOSIP CHO	Viral-derived	SOSIP 293F	SOSIP CHO
Oligomannose	M5	2.7	8.1	15	0.3	6.9	14
	M6	4.5	3.7	4	1.4	4.8	4
	M7	7.7	6.8	5	2.0	7.0	4
	M8	13.6	21.0	14	0.0	3.7	3
	M9	21.4	23.5	35	0.0	0.0	1
	TOTAL	49.9	63.1	73	3.7	22.4	26
Sialic Acids	α2-3	9.6	3.3	N.D.	9.3	4.2	N.D.
	α2-6	6.6	2.1	N.D.	16.9	3.4	N.D.
	TOTAL	16.3	5.3	N.D.	26.1	7.5	N.D.

Table S2: Quantification of N-linked glycans using exoglycosidase digests and subsequent HPLC analysis of gp120 and gp41 glycans from the BG505.T332N-LAI virion and 293F-derived BG505 SOSIP.664. Values represent percentages of total glycan population. CHO data reproduced fromDey et al. (2018). ‘Sialic acids’ refers to sialylated glycans. Related to Figure 1.
